# The clinical efficacy of using autologous platelet-rich plasma in total hip arthroplasty: A retrospective comparative study

**DOI:** 10.1097/MD.0000000000012451

**Published:** 2018-10-05

**Authors:** Xi Wang, Jianbin Ma, Zhiyuan Wang, Lin Xiao

**Affiliations:** Honghui Hospital, Xi’an Jiaotong University College of Medicine, Xi’an, Shaanxi Province, China.

**Keywords:** intra-articular platelet-rich plasma, retrospective comparative study, total hip arthroplasty

## Abstract

This study evaluated whether intra-articular platelet-rich plasma (PRP) might prevent postoperative bleeding in total hip arthroplasty (THA).

In this study, 260 hips that underwent THA were evaluated randomly by paramedical staffs, 130 of which involved the intraoperative use of PRP, and 130 of which served as control group. Postoperative blood loss (drain bag volume), estimated blood loss, and change in hemoglobin (Hb) at day 1, 2, 4, and 7 were analyzed, respectively.

PRP-treated group had a significant decrease in mean postoperative blood loss (92.6 ± 168.2 mL) compared to control group (682.3 ± 185.5 mL, *P* < .01). The mean postoperative estimated blood loss (526.1 ± 236.1 mL) in the PRP-treated group was significantly less than that in the control group (629.2.2 ± 142.3 mL, *P* < .01). There was a statistically significant difference in Hb value (mg/dL) at day 1, 2, 4, and 7 (−1.35 vs −1.98, −1.59 vs −2.52, −1.96 vs −2.82, and −1.76 vs −2.47, *P* < .05).

We found a significant reduction in postoperative blood loss (drain bag volume), estimated blood loss, and change in Hb after the use of autologous platelet gel in patients of THA, and PRP appears to be effective in reducing postoperative bleeding in THA.

## 1 Introduction

Orthopedic surgery, especially total hip arthroplasty (THA), strict postoperative bleeding requirements, because postoperative bleeding will affect the limb functional recovery and function.^[1]^ Postoperative bleeding may lead to surgical failure, and even physical disability. Therefore, how to reduce bleeding after THA becomes a clinical focus.^[2,3]^

Platelet-rich plasma (PRP) is a platelet concentrate extracted from autologous blood by centrifugation. Nearly 10 years, PRP has been applied to a variety of disciplines especially in Europe and the United States, because it can promote bone and soft tissue repair and is easy to be obtained. Many clinical research reports that PRP can accelerate fracture healing, promote wound recovery, reduce postoperative complications, and promote postoperative functional recovery.^[4,5]^

PRP contains platelets, growth factors, leukocytes, fibronectin, and some other bioactive components.^[6]^ The primary role of platelets in PRP is its effect on tissue repair. Experiments show that platelets can be adhered to the blood vessel wall and inserted into the endothelial cells or into the cytoplasm of endothelial cells to repair the vascular endothelium and to maintain the integrity of the endothelium. Recent studies have shown that platelets also play an important role in angiogenesis and can promote vascular regeneration by releasing antigenic regulatory factors, such as vascular endothelial growth factor and fibroblast growth factor. Leukocytes can help the body to clear the local pathogens, enhance the ability of local anti-infection, and also help the body to remove the local necrotic tissue, significantly speed up the local tissue damage repair rate, which effectively promotes the repair of local tissue damage.^[7]^

There are many useful ingredients, including fibronectin, platelet-reactive protein, osteoproteins, vitronectin, etc., in PRP, in addition to platelets, leukocytes, and fibronectin. They play an important role in wound infection, promoting tissue repair and increasing neovascularization.

Hip arthroplasty is often associated with significant perioperative complications including blood loss necessitating blood transfusions, which can lead to multiple adverse reactions, infection transmission, and longer hospital stay.^[8–10]^ PRP is rich in platelet-derived growth factor (PDGF), insulin-like growth factor, vascular endothelial growth factor, epidermal growth factor, transforming growth factor beta 1/2, and other growth factors. These growth factors play an important role in the regulation of cell regeneration, differentiation, and extracellular matrix synthesis.^[7]^ PRP can provide the healing environment required for wound healing, through the release of growth factors continued acted on tissues; thus, promoting wound healing and reducing bleeding.^[11]^

In this study, we used PRP for THA, and evaluated the efficiency of PRP.

## 2 Methods

### 2.1 Patient selection criteria

Inclusion criteria: (1) unilateral femoral neck fracture; (2) age 60-75 years; (3) initial artificial total hip arthroplasty. Exclusion criteria: (1) combined with other parts of the fracture; (2) preoperative limb deep vein thrombosis or combined with other medical diseases administration with aspirin and other antiplatelet drugs, or the presence of blood system diseases affect the coagulation function; (3) physical condition cannot tolerate artificial total hip Arthroplasty.

A total of 260 hips were eligible for inclusion in the study. The patient number was randomly divided into 2 groups. The evens were PRP-treated group and odds were the control group, each group of 130 cases. Patients are willing to participate and sign informed consent. This study was approved by XXX Hospital Ethics Committee.

### 2.2 Preparation of PRP

PRP was prepared as previously reported. Before surgery, 8 mL ACD-A solution was extracted by 60 mL syringe with sterile conditions; and then, extracted the patient 52 mL venous blood. The extraction was mixed well and then transferred into a special centrifuge tube. Centrifuging is performed at a radius of 15 cm, 2400 rpm/min for 12 min. Finally, PRP was stocked in a 12 mL syringe.

### 2.3 Surgery method

Two groups of surgery were completed by the same group of doctors. The same prosthesis was used in THA. After general anesthesia, patients were with lateral position. Posterolateral approach was performed and the incision length was 12 to 15 cm, which is in accordance with conventional artificial THA operation. The appropriate prosthesis was implanted. Traction under the reset, active hip and verify no dislocation tendency. Saline was sued to flush incision and then complete hemostasis. PRP was injected into the joint cavity in the PRP-treated group and the control group was injected with the same dose of saline. We placed the drainage tube and closed the wound layer by layer.^[12]^

### 2.4 Postoperative treatment and efficacy evaluation indicators

Two groups of postoperative treatment were consistent. We remove the drainage tube 48 h after operation. After extubation, low molecular weight heparin calcium was administrated to prevent deep vein thrombosis. Postoperative limb abduction median position was sustained for 2 weeks. Quadriceps isometric contraction exercise was performed on the bed 2 days after the operation. Out of bed exercise was conducted 3 to5 days after the operation. Postoperative bleed loss volume and hemoglobin (Hb) value 1, 2, 4, and 7 days after the operation were recorded.

### 2.5 Statistical analysis

SPSS 19.0 statistical software was used for statistical analysis. The data were expressed as mean ± standard deviation, and the paired *t* test was used for before and after the operation. The *t* test was used for the comparison between the two groups. The significant difference level was *P* < .05.

## 3 Results

Between January 2015 and February 2017, 260 patients underwent THA under the same surgeon. One-hundred thirty patients were treated with PRP and 130 patients served as untreated control.

PRP treated group contains 68 males and 62 females. The age of PRP-treated group ranges from 61 to 75 years. The mean age is 67.5 years old. The onset sides of fracture of PRP-treated group were 52 cases on the left and 78 cases on the right. The causes of injury were 45 cases of falls, 52 cases of traffic accidents, and 33 cases of high-energy injury. The time after the injury to the hospital was for 1 h to 12 days and the median time was 4 days. Fracture classification: Garden III type was 72 cases and IV type was 58 cases. The control group contains 70 males and 60 females. The age of control group ranges from 60 to 73 years. The mean age is 66.7 years old. The onset sides of fracture of control group were 59 cases on the left and 71 cases on the right. The injury causes were 50 cases of falls, 70 cases of traffic accidents, and 10 cases of high-energy injury. The time after the injury to hospital was for 2 h to 11 days and the median time was 3 days. Fracture classification: Garden III type was 69 cases and IV type was 61 cases. Two groups of patients were closed fractures, without nerve, blood vessels, and other combined injury, but with varying degrees of hip swelling, external rotation deformity, tenderness and limited activity, and other symptoms. There were no significant differences in sex, age, course of disease, cause of injury, and type of fracture between the two groups (*P* > .05) (Table 1).

**Table 1:**
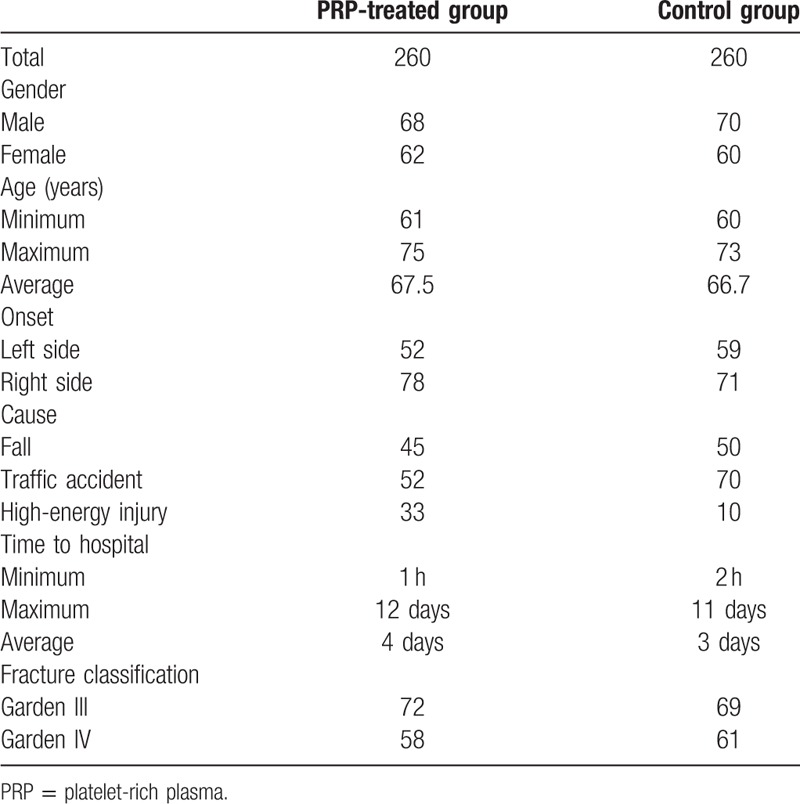
General information of the patients.

**Figure 1:**
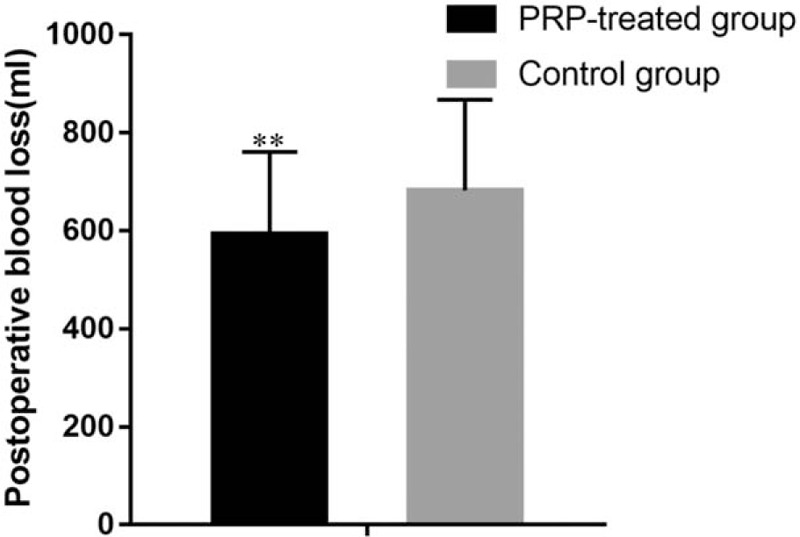
Postoperative blood loss of PRP-treated group and control group. ^∗^*P* < .05 and ^∗∗^*P* < .01. PRP = platelet-rich plasma.

**Figure 2:**
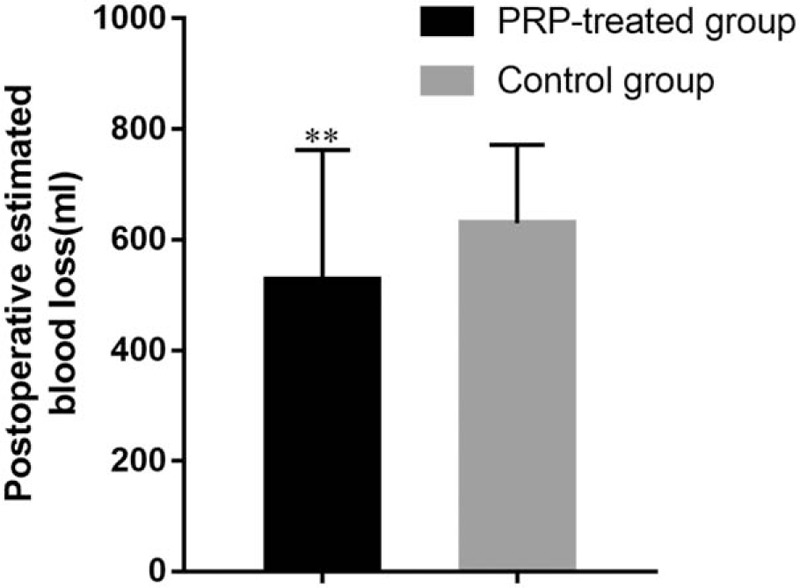
Postoperative estimated blood loss of PRP-treated group and control group. ^∗^*P* < .05 and ^∗∗^*P* < .01. PRP = platelet-rich plasma.

The mean postoperative blood loss of 592.6 ± 168.2 mL in the PRP-treated group was significantly less than that in the control group (682.3 ± 185.5 mL, *P* < .001). The mean postoperative estimated blood loss of 526.1 ± 236.1 mL in the PRP-treated group was significantly less than that in the control group (629.22 ± 142.3 mL, *P* < .01) (Figs. 1 and 2).

**Figure 3:**
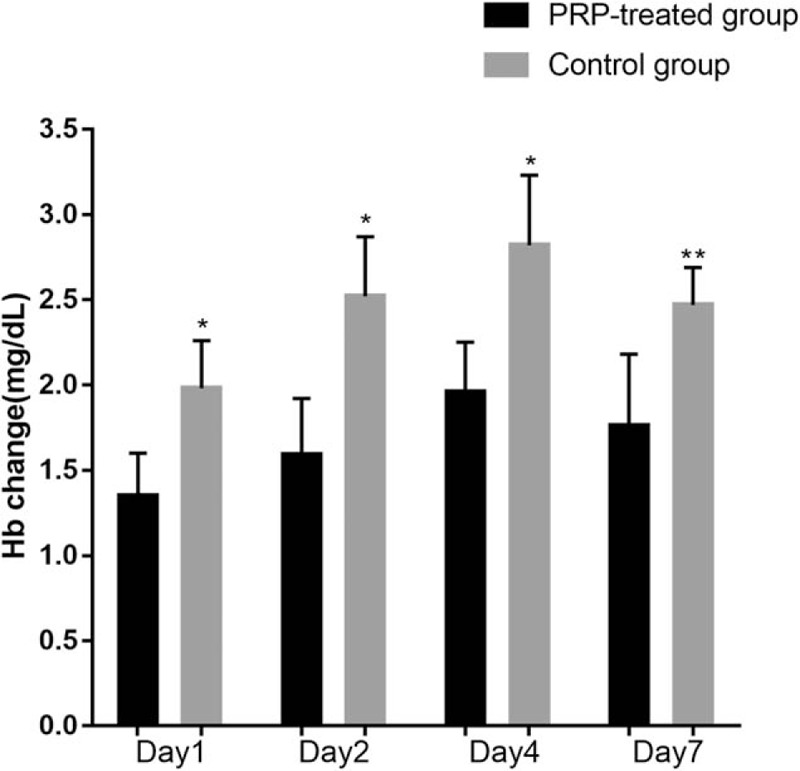
The Hb change of different time after operation compared with preoperation. ^∗^*P* < .05 and ^∗∗^*P* < .01. Hb = Hemoglobin.

The mean change in Hb value (mg/dL) from baseline was −1.35 in the PRP-treated group and −1.98 in the control group at postoperative 1 day (*P* < .05), −1.59 in the PRP-treated group and −2.52 in the control group at postoperative day 2 (*P* < .05), −1.96 in the PRP-treated group and −2.82 in the control group at postoperative day 4 (*P* < .05), and −1.76 in the PRP-treated group and −2.47 in the control group at postoperative day 7 (*P* < .01). At each time point, there were significant differences in the Hb levels between the PRP-treated group and the control group (Fig. 3).

## 4 Discussion

Postoperative blood loss and wound healing is the result of the synergistic effect of repairing cells, inflammatory cells, extracellular matrix, and growth factors, and is a complex process of regulating and interacting with different growth factors.^[6,7,13]^ Studies have shown that the effect of multiple growth factors is better than that of single growth factor, which may be related to a variety of factors, which are related to multiple stages of the healing process.^[14,15]^

PRP is a platelet-rich plasma concentrate extracted from whole blood, containing high concentrations of platelets, fibrin, and white blood cells. Studies have shown that PRP can release granules through platelet α-particles, thereby releasing a series of cytokines to play a biological effect, effectively promote the repair of musculoskeletal system injury, which has been widely clinically used in the treatment of refractory wounds, soft tissue defects, and delay fracture healing and nonunion.^[16,17]^ PRP activation can form a natural growth factor-rich fibrin stent, can effectively load cells, build tissue engineering repair materials; also can shrink the wound, with coagulation; release of growth factors combined with the target cells membrane transmembrane receptors, thereby inducing activation of endogenous protein signaling, further inducing gene expression within target cells, promoting cell proliferation, collagen synthesis, and matrix formation.^[18,19]^ In addition, PRP can significantly accelerate cellular metabolic activity, reduce cell apoptosis, and stimulate immature cell synthesis of collagen fibers. In this study, postoperative bleeding in the PRP-treated group was significantly lower than the control group, suggesting that PRP has procoagulant effect, can promote wound repair and help patients with postoperative joint function exercise and rehabilitation.^[20–22]^

PRP contains many bioactive components to play biological functions. PDGF is mainly synthesized by megakaryocytes and stored in alpha granules of platelets. Other cells can also produce PDGF, such as macrophages, endothelial cells, fibroblasts, myoblasts, glioma cells, and glioblastoma, and other tumor cell lines after appropriate stimulation.^[19]^ PDGF can induce cells to do something like synthesis of DNA, activation of tyrosine kinases, upregulation of arachidonic acid, PGI2 and PGE3 release, activation of adenylate cyclase, activation of serine–threonine kinase, induction of gene transcription, and alter cell surface activity. Some studies indicated that PDGF induced cell gene expression alteration. PDGF not only can promote cell proliferation, but also increase the synthesis of collagen and noncollagen components.^[23]^

In addition, due to white blood cells and platelets in the centrifugal settlement speed similar to the preparation of PRP process of platelet and leukocyte sedimentation in the same level, PRP contains high concentrations of white blood cells.^[12,24,25]^ Leukocytes can help the body to clear the local pathogens, enhance the local anti-infective ability, but also help the body to remove the local necrotic tissue, to speed up the local tissue repair rate, thereby promoting local tissue damage repair. In addition, leukocytes chemotaxis by platelet secretion of growth factors can also secrete growth factors itself, which was directly involved in tissue repair.^[26,27]^ PRP also has many useful ingredients, including fibronectin, platelet-reactive protein, osteoconductive protein, vitreous connexin, etc. These components play an important role in preventing blood loss and wound infection, promoting tissue repair and increasing the formation of neovascularization. The PRP-treated group of adverse reactions rate of 0, which lower than the control group of 1.3%, indicating that PRP in clinical use will not increase the risk of adverse reactions.^[28,29]^

In this study, PRP was used in THA for reducing postoperative blood loss with the following advantages:

1. Autologous PRP is without immune rejection reaction; thus, there is no risk of allogeneic transplantation rejection diseases.
2. PRP can not only prevent platelet loss, but also adhere to tissue defects, so that platelets can locally long-term secret growth factors to maintain a high growth factor concentration. This avoids the current widely used in clinical liquid recombinant growth factor reagents, which is easily lost in the wound and evaporate in the short time.
3. As the white blood cells, monocytes, and platelets in the blood sedimentation coefficient is similar, so the preparation of the PRP by centrifugation also contains some white blood cells and monocytes, which can prevent infection.
4. The production is harmless to patients and is simple, fast and easy to learn and master. The process can be completed in the operating room and surgery at the same time. This can reduce the intermediate links and the pollution risk. Therefore, it may be able to effectively reduce medical costs and promote wound healing.

## Author contributions

**Data curation:** Xi Wang

**Formal analysis:** Xi Wang and Lin Xiao

**Funding acquisition:** Xi Wang and Lin Xiao

**Investigation:** Zhiyuan Wang

**Methodology:** Lin Xiao

**Resources:** Lin Xiao

**Software:** Jianbin Ma and Lin Xiao

**Supervision:** Jianbin Ma and Zhiyuan Wang

**Validation:** Zhiyuan Wang and Lin Xiao
